# Editorial: Postnatal brain development in moderate and late preterm infants: challenges and context-relevant interventions

**DOI:** 10.3389/fpsyg.2024.1511981

**Published:** 2024-11-04

**Authors:** Antonio Pereira, Sheila Andreoli Balen, Silvana Alves Pereira

**Affiliations:** ^1^Laboratory of Signal Processing, Institute of Technology, Federal University of Pará, Belém, PA, Brazil; ^2^Speech, Language and Hearing Department, Federal University of Rio Grande do Norte, Natal, RN, Brazil; ^3^Department of Physical Therapy, Federal University of Rio Grande do Norte, Natal, RN, Brazil

**Keywords:** preterm, late preterm, neurodevelopment, postnatal care, language, motor development

## Introduction

Advancements in neonatal care have greatly improved the survival rates of preterm infants, yet long-term neurodevelopmental outcomes remain a significant concern. While very preterm and very-low-birthweight infants are often considered the highest-risk groups, moderate and late preterm infants also face unique neurodevelopmental challenges that can persist well-beyond the neonatal period. The eight studies featured in this Research Topic explore various aspects of postnatal brain development, offering valuable insights into targeted interventions that can enhance cognitive, motor, and sensory development in these infants.

This Research Topic of studies emphasizes the critical role of early interventions to mitigate neurodevelopmental delays. Such insights are important in both high-resource and low-resource settings, where socioeconomic factors significantly influence outcomes. Most preterm births occur in developing countries where preterm infants are at higher risks of neurodevelopmental complications due to insufficient medical and social support (WHO, 2018). Interventions tailored to these settings, as explored in the studies presented here, are crucial for improving developmental trajectories and providing equitable healthcare access.

## Parental engagement and lexical-semantic networks

Ragó et al. investigate how preterm birth affects the organization of the early lexical-semantic network in infants. Their use of eye-tracker technology revealed that while full-term infants more efficiently organize these networks, preterm infants also show stable, though slightly delayed, patterns of organization. This study underscores the importance of language-rich environments for preterm infants, advocating for parental involvement in promoting early language development to mitigate any delays associated with premature birth.

## Cognitive development in school-aged preterm infants

Lacalle et al. conducted a systematic review examining the cognitive development of school-aged preterm infants, focusing on IQ. Across 40 studies involving over 5,000 preterm children, preterm infants consistently scored lower on IQ tests compared to full-term peers. Socioeconomic factors, family education levels, and early health interventions were identified as key influences on cognitive outcomes, stressing the need for tailored strategies to bridge this developmental gap. Targeted cognitive interventions, particularly in underprivileged environments, are essential for bridging these developmental gaps, as environmental factors significantly shape cognitive outcomes during school years.

## Early motor and cognitive interventions

Sampaio et al. conducted a clinical trial on the benefits of tummy time (TT) for preterm infants in low-income settings. The study showed that TT significantly improved motor skills such as head elevation and cognitive development. The intervention is simple and cost-effective, providing parents and caregivers in low-income settings where more advanced therapies may be unavailable with an accessible tool to enhance their infants' developmental growth.

## Motor development and reaching behavior

França et al. explored reaching behavior in late and very preterm infants, finding that very preterm infants displayed delayed acquisition of reaching skills. The study highlights the importance of interventions targeting fine motor skills in preterm infants to ensure they do not fall behind in achieving critical motor milestones, which are foundational for cognitive and physical development.

## Auditory and speech processing

Ribas-Prats et al. presented a pilot study investigating auditory processing using frequency-following response (FFR) recordings in late preterm infants. The findings revealed that preterm infants have delayed neural encoding of speech sounds, which may have long-term consequences for language development. Early screening and interventions should thus be a high priority, since addressing auditory impairments during infancy can lead to better speech and language outcomes, particularly in environments where speech and sound play critical roles in cognitive development.

## Congenital conditions and auditory and language impacts

Ferreira et al. conducted a review on the auditory consequences of congenital toxoplasmosis, often associated with prematurity. The study found an increased risk of hearing loss in preterm infants with toxoplasmosis, emphasizing the need for early and comprehensive auditory screening to prevent language impairments, as undiagnosed auditory deficits can severely affect language development and social integration.

## Inflammatory diseases and neurodevelopmental disorders

Huang et al. examined the link between type 2 inflammatory diseases (such as asthma and atopic dermatitis) and neurodevelopmental disorders in low-birth-weight children, many of whom were born preterm. Their study found a strong association between these diseases and conditions such as autism spectrum disorder (ASD) and attention deficit hyperactivity disorder (ADHD), underlining the importance of early immune and neurodevelopmental monitoring to improve outcomes.

## Interdisciplinary approaches to improve outcomes

McCarty et al. explored an interdisciplinary model for eye examinations in preterm infants at risk for retinopathy. The study demonstrated that collaborative care reduced stress during exams, leading to improved heart rate recovery and better neurodevelopmental outcomes. These results reinforce the need of interdisciplinary approaches that combine medical, psychological, and developmental expertise as essential for addressing the complex needs of preterm infants in neonatal care settings.

## Conclusion

The studies in this Research Topic offer a comprehensive view of the challenges and opportunities in optimizing neurodevelopment in preterm infants. From cognitive and motor interventions to addressing sensory deficits and neurodevelopmental disorders, early, targeted interventions are essential. Given the heavy burden of preterm births in developing countries, where the availability of care is limited, these findings are crucial for improving the outcomes of preterm infants globally. By addressing the unique needs of preterm infants in diverse settings, we can improve their developmental trajectories and enhance their long-term health outcomes.
